# The combination of atrial fibrillation and small vessel disease score worsen spontaneous intracerebral hemorrhage outcomes

**DOI:** 10.3389/fneur.2025.1682520

**Published:** 2025-10-29

**Authors:** Yuki Hamada, Takeo Sato, Hideki Matsuoka, Yuka Tsuchimochi, Kaishi Kukihara, Yutaro Kawabata, Kana Iwamoto, Go Takaguchi, Yujiro Higuchi, Hiroshi Takashima

**Affiliations:** ^1^Department of Strokology, Stroke Center, National Hospital Organization Kagoshima Medical Center, Kagoshima, Japan; ^2^Department of Neurology and Geriatrics, Kagoshima University Graduate School of Medical and Dental Sciences, Kagoshima, Japan

**Keywords:** atrial fibrillation, intracerebral hemorrhage, small vessel disease, functional outcome, brain-cardiac interaction

## Abstract

**Background:**

This study aimed to examine whether the presence of atrial fibrillation (AF), severity as judged by total small vessel disease (SVD) score, or a combination of these is associated with prognosis and hematoma volume of spontaneous intracerebral hemorrhage (sICH).

**Methods:**

This retrospective analysis investigated 608 patients who were admitted within 7 days of ICH onset between July 2012 and November 2023. The primary outcome was prognosis at 3 months after onset according to the modified Rankin Scale (mRS) score. Associations of AF, SVD and a combination of these with outcomes and hematoma volume were examined using univariate analyses, logistic regression analysis, and multiple regression analysis.

**Results:**

A total of 608 consecutive patients with sICH were screened, and 330 patients were finally included in the analysis after applying the inclusion and exclusion criteria. Among the 330 patients analyzed, 145 (43.9%) experienced poor outcomes (mRS score 4–6). The presence of AF was independently associated with poor outcome (adjusted OR 4.93, 95% CI 1.72–16.1; *p* = 0.002), whereas total SVD score alone was not. Multiple regression analysis with estimated hematoma volume also showed a significant association with AF (standard partial regression coefficient 0.16; *p* = 0.033), but not with total SVD score. However, a linear trend was observed between the combination of AF and total SVD score severity and poor outcome (total SVD score 0–1 without AF: aOR 1.00, total SVD score 2–4 without AF: 1.44, total SVD score 0–1 with AF: 4.26, total SVD score 2–4 with AF: 7.57; *p* for trend = 0.002). Multiple regression analysis using estimated hematoma volume also showed a synergistic effect of AF and total SVD score severity (standardized regression coefficient 0.14; *p* = 0.032).

**Conclusion:**

The presence of AF was associated with poor 3-month outcomes and increased estimated hematoma volume in sICH patients, whereas total SVD score alone was not. The effects of AF and SVD together appear to contribute synergistically to worsened prognosis and increased hematoma volume in sICH patients.

## 1 Introduction

Spontaneous (non-traumatic) intracerebral hemorrhage (sICH) accounts for approximately 10–15% of all strokes and is associated with high morbidity and mortality rates (1, 2). In 2016, the global age-standardized incidence of sICH was reported as 22.2 cases per 100,000 person-years, and hemorrhagic stroke was associated with a 30-day mortality rate of up to 40% (3, 4). The prognosis after sICH is related to the initial hematoma volume and subsequent expansion, which may be worsened by antithrombotic therapy, including oral anticoagulants (OACs) and antiplatelet agents (PAs) (5–9).

Notably, OAC use is a significant cause of sICH and is closely related to the presence of atrial fibrillation (AF), which is frequently observed in sICH patients. In the AF population, the reported incidence of sICH is 0.31–0.78 per 100 person-years (10, 11), and individuals with AF in general have a 1.5–1.9 times higher risk of mortality compared to those without AF (12). This increased risk is mainly due to cardiovascular diseases such as heart failure and ischemic stroke, but OAC-related sICH has also been associated with high mortality in numerous studies (13). Further, regarding the two types of OACs—vitamin K antagonists and direct OACs—many reports have found no significant difference in short-term prognosis among sICH patients, leaving uncertainty as to which contributes more to poor outcomes (14, 15). Overall, these studies have suggested that sICH and AF may synergistically influence poor prognosis, but no direct causal relationship between AF and sICH outcomes has been identified. Moreover, acute spontaneous lobar ICH is often characterized by a distinct clinical profile and a more severe early prognosis compared with deep subcortical ICH, and non-hypertensive mechanisms are more likely to predominate in lobar hemorrhages (16).

In general, sICH results from cerebral small vessel disease (SVD), with arteriosclerosis and cerebral amyloid angiopathy as the major underlying pathologies (17). In this study, we focused on SVD, a condition associated with high risks of stroke and poor prognosis. SVD gradually progresses with aging and arteriosclerosis, increasing stroke risk and worsening outcomes (18). If the presence of AF and the severity of SVD influence poor sICH outcomes, preventing SVD itself may contribute to reducing sICH incidence and improving prognosis in AF patients. We therefore hypothesized that AF and the severity of SVD are associated with poor sICH prognosis and investigated the relationship with prognostic indicators such as hematoma volume. In addition, we examined whether the combination of AF and severe SVD results in an exponential increase in poor sICH outcomes.

## 2 Subjects and methods

### 2.1 Study population and study protocol

Patients were selected from the prospective and retrospective acute stroke database, the “Kagoshima Medical Center Stroke Database Registry,” at Kagoshima Medical Center, a secondary medical facility located in Kagoshima City, Kagoshima Prefecture. A continuous retrospective study was conducted on all 608 ICH patients admitted between July 2012 and November 2023. The inclusion criteria were as follows: (1) admission within 7 days of onset; (2) history of AF or new diagnosis of AF during hospitalization; (3) availability of total SVD score data; and (4) recorded modified Rankin Scale (mRS) score at 3 months post-onset. The exclusion criteria were: (1) causes of intracerebral hemorrhage other than spontaneous occurrence, such as aneurysm, vascular malformations, brain tumors, moyamoya disease, or shunt-related disorders (e.g., dural arteriovenous fistula and arteriovenous malformation) detected on initial imaging; (2) ICH occurring after treatment for acute ischemic stroke (intravenous thrombolysis or mechanical thrombectomy); or (3) traumatic ICH. All patients were consecutively extracted from our institutional stroke registry without any age restrictions, and the age range of the included patients was 16–95 years.

### 2.2 Data collection

Data were obtained from our institution’s proprietary database and hospitalization records, including the following variables: sex, age, pre-onset mRS score, body mass index, systolic and diastolic blood pressures on admission, total National Institutes of Health Stroke Scale score on admission, wake up stroke, current smoking status, excessive alcohol consumption [equivalent to ≥3 servings of sake/day or ≥60 g of pure alcohol/day (19)], history of any stroke [ischemic stroke, intracerebral hemorrhage, or transient ischemic attack (TIA)], history of anticoagulant use prior to onset, type of anticoagulant used before onset, history of antiplatelet use before onset, history of hypertension, diabetes mellitus [defined as a pre-hospitalization diagnosis, use of glucose-lowering medication before admission, pre-hospitalization diagnosis without treatment, or hemoglobin A1c (National Glycohemoglobin Standardization Program) ≥6.5% on admission], dyslipidemia (defined as pre-hospitalization diagnosis, use of lipid-lowering agents before admission, pre-hospitalization diagnosis without treatment, or low-density lipoprotein cholesterol ≥140 mg/dL on admission), presence of atrial fibrillation (either pre-hospitalization history or new diagnosis during hospitalization), and laboratory test results on admission [hemoglobin, hematocrit, blood urea nitrogen, creatinine, uric acid, low-density lipoprotein cholesterol, triglycerides, glycosylated hemoglobin (National Glycohemoglobin Standardization Program), brain natriuretic peptide, and D-dimer]. Among patients with AF, cardiac comorbidities were further categorized according to the classification proposed by Pujadas Capmany et al. (20). Structural cardiac disorders associated with arrhythmia, including valvular heart disease, cardiomyopathy, ischemic heart disease, left ventricular hypertrophy, and left ventricular dysfunction, were identified, as well as isolated atrial dysrhythmia without structural abnormalities. In addition, imaging data obtained from computed tomography (CT) performed at our hospital included hematoma location, estimated hematoma volume (21), presence of hematoma expansion (22), presence of intraventricular hemorrhage, and laterality of cerebral lesions. Magnetic resonance imaging (MRI) data included the presence of white matter hyperintensities (WMHs) (23), location and number of cerebral microbleeds (CMBs) (24), enlarged perivascular space (EPVS), severity (25), presence and location of chronic lacunar infarcts (26), and total SVD score (25–27). Data on the use of reversal agents for anticoagulation-associated hemorrhage (idarucizumab, andexanet alfa, lyophilized human prothrombin complex concentrate), surgical treatment for intracerebral hemorrhage, type of surgical intervention, mRS score at 3 months post-onset, and 3-month mortality were also collected.

The primary outcome was defined as a poor outcome (mRS score 4–6) at 3 months post-onset. This database study included patients admitted to the neurology and vascular medicine department at our hospital for treatment or examination, and additional surgical interventions were performed for patients deemed to require them at a later stage.

### 2.3 Total SVD score

The following SVD markers were used in the present study: presence of old lacunae, WMH scale [scored separately as periventricular hyperintensity (PVH) and deep and subcortical WMH (DSWMH)] (28), number of CMBs, and severity of EPVS (29). An old lacuna was defined as a round or ovoid hypointense area with hyperintense rim on fluid-attenuated inversion recovery (FLAIR) imaging or as a hyperintense area on T2-weighted imaging (T2WI) of 3–15 mm (30) in the following sites: deep subcortical white matter including the corona radiata, caudate head, lentiform, posterior limb and genu of the internal capsule, thalamus, or brainstem including the midbrain, pons and medulla (25). PVH and DSWMH were evaluated using the Fazekas grading scale, as modified by Shinohara (separately rating the deep and periventricular regions on a scale of 0–4) for lesions showing hyperintensity on FLAIR imaging (28, 29). CMBs were defined as small voids showing an area of hypointensity 2–10 mm in diameter that were visible on gradient-recalled echo T2*-weighted MRI (T2*-GRE) (24). EPVS was defined as a linear hyperintensity on T2WI or linear hypointensity on FLAIR in the basal ganglia and centrum semiovale (23). The severity of EPVS in the basal ganglia was graded according to the number of EPVS observed, as follows: grade 0, no EPVS; grade 1, 1–10 EPVS; grade 2, 11–20 EPVS; grade 3, 21–40 EPVS; and grade 4, ≥41 EPVS (31). Total SVD score was calculated as the sum of points for each of the following four imaging findings (each scored as 1 point): ≥1 old lacunae, presence of WMH (periventricular: Fazekas grade 3 and/or deep white matter: Fazekas grade 2–3), ≥1 CMBs, and ≥11 basal ganglia EPVS (EPVS grade ≥2). The maximum (most severe) score was 4.

### 2.4 Imaging protocols

At our hospital, patients with suspected stroke basically undergo MRI on admission. After that, patients with ICH confirmed on MRI undergo CT immediately after MRI. Patients with ICH routinely undergo a second CT more than 24 h after admission.

CT platforms and parameters used in this study were SOMATOM Sensation 64 (Siemens Healthineers, Germany; 120 kVp, 385 mAs, and slice thickness 5 mm), SOMATOM Definition Flash (Siemens, 120 kVp, 410 mAs or reference 460 mAs, and slice thickness 5 mm), or SOMATOM Definition AS (Siemens, 120 kVp, 385 mAs or reference 460 mAs, and slice thickness 5 mm).

The MRI platforms used in this study were as follows: (1) MAGNETOM Avanto (Siemens) at 1.5 T. Sequence parameters for T2 imaging were: TR (repetition time), 4,480 or 5,120 ms; TE (echo time), 87 ms; matrix, 320 × 320; and FOV (field of view), 220 mm. Sequence parameters for FLAIR imaging were: TR, 8,000 ms; TE, 110 ms; matrix, 256 × 256; and FOV, 210 mm. Sequence parameters for T2* imaging were: TR, 600 or 1,890 ms; TE, 25 or 38 ms; matrix, 224 × 256; and FOV, 220 mm; (2) MAGNETOM Avanto Dot (Siemens) at 1.5 T. Sequence parameters for T2 imaging were: TR, 4,300 or 8,530 ms; TE, 87 ms; matrix, 320 × 320; and FOV, 220 mm. Sequence parameters for FLAIR imaging were: TR, 8,000 ms; TE, 103, 110 or 127 ms; matrix, 256 × 256; and FOV, 220 mm. Sequence parameters for T2* imaging were: TR, 647 ms; TE, 21.9 or 22 ms; matrix, 224 × 256; and FOV, 220 mm; (3) Ingenia (Philips, Netherlands) at 3.0 T. Sequence parameters for T2 imaging were: TR, 3,000 ms; TE, 90 ms; matrix, 512 × 512; and FOV, 220 mm. Sequence parameters for FLAIR imaging were: TR, 11,000 ms; TE, 140 ms; matrix, 512 × 512; and FOV, 220 mm. Sequence parameters for T2* imaging were: TR, 587, 622 or 649 ms; TE, 16.11 ms; matrix, 512 × 512; and FOV, 220 mm.

### 2.5 Statistical analysis

Statistical analysis was performed using JMP version 15 (SAS Institute, Cary, NC, United States). Clinical characteristics were divided into favorable and poor outcome groups. In univariate analyses, categorical variables were compared using the *χ*^2^ test or Fisher’s exact test, while continuous variables were analyzed using the Mann–Whitney test.

Further, logistic regression analysis was performed with poor outcome as the dependent variable, and AF, total SVD score, and prespecified factors related to the prognosis and hematoma volume of intracerebral hemorrhage (age, sex, history of any stroke, presence of OACs, presence of oral PAs, systolic blood pressure on admission, and estimated hematoma volume) as independent variables.

In addition, multiple regression analysis was conducted with estimated hematoma volume as the dependent variable, adjusted for the following prespecified factors: age, sex, history of any stroke, presence of OACs, presence of oral PAs, and systolic blood pressure on admission. To avoid multicollinearity among independent variables, the variance inflation factor (VIF) was checked, and variables with high correlation (VIF ≥10) were excluded.

Moreover, previous studies have reported that a total SVD score of 2–4 is associated with poor outcomes after ischemic stroke (32). We therefore divided the total SVD score into two categories (0–1 or 2–4) and evaluated the impact of the combination with the presence or absence of AF on ICH outcomes.

To confirm the linear relationship between AF, which was significantly associated with poor outcomes in the aforementioned multivariate analysis, and SVD, we classified patients into four groups and assigned indicator variables to each group (total SVD score 0–1 without AF: 1; total SVD score 2–4 without AF: 2; total SVD score 0–1 with AF: 3; and total SVD score 2–4 with AF: 4).

We then built another logistic regression model that included the three indicator variables (Indicator variables 2–4) associated with poor outcome in reference to Indicator variable 1. This model was also adjusted for the prespecified factors associated with poor outcome mentioned above. In addition, we calculated the *p* for trend.

To examine the impact of the combination of AF and total SVD score on estimated hematoma volume, we conducted a multiple regression analysis that included the previously mentioned indicator variables as ordinal variables. This analysis was adjusted for the following prespecified factors related to hematoma volume: age, sex, history of any stroke, presence of OACs, presence of oral PAs, systolic blood pressure on admission, and presence of subcortical hemorrhage.

The mean estimated hematoma volume for each indicator variable was also calculated. In all statistical analyses, values of *p* < 0.05 were considered statistically significant. As this study analyzed consecutive cases extracted from our institutional stroke registry, no *a priori* sample size calculation was performed.

### 2.6 Ethics approval

This study was approved by the Ethics Committee of Kagoshima Medical Center (Approval No. 2024-03). The committee waived the requirement for patient consent and instead provided those patients from whom data were collected with the opportunity to opt out of the study. The study protocol adhered to the ethical guidelines of the Declaration of Helsinki, as revised in 1975.

## 3 Results

**Figure 1:**
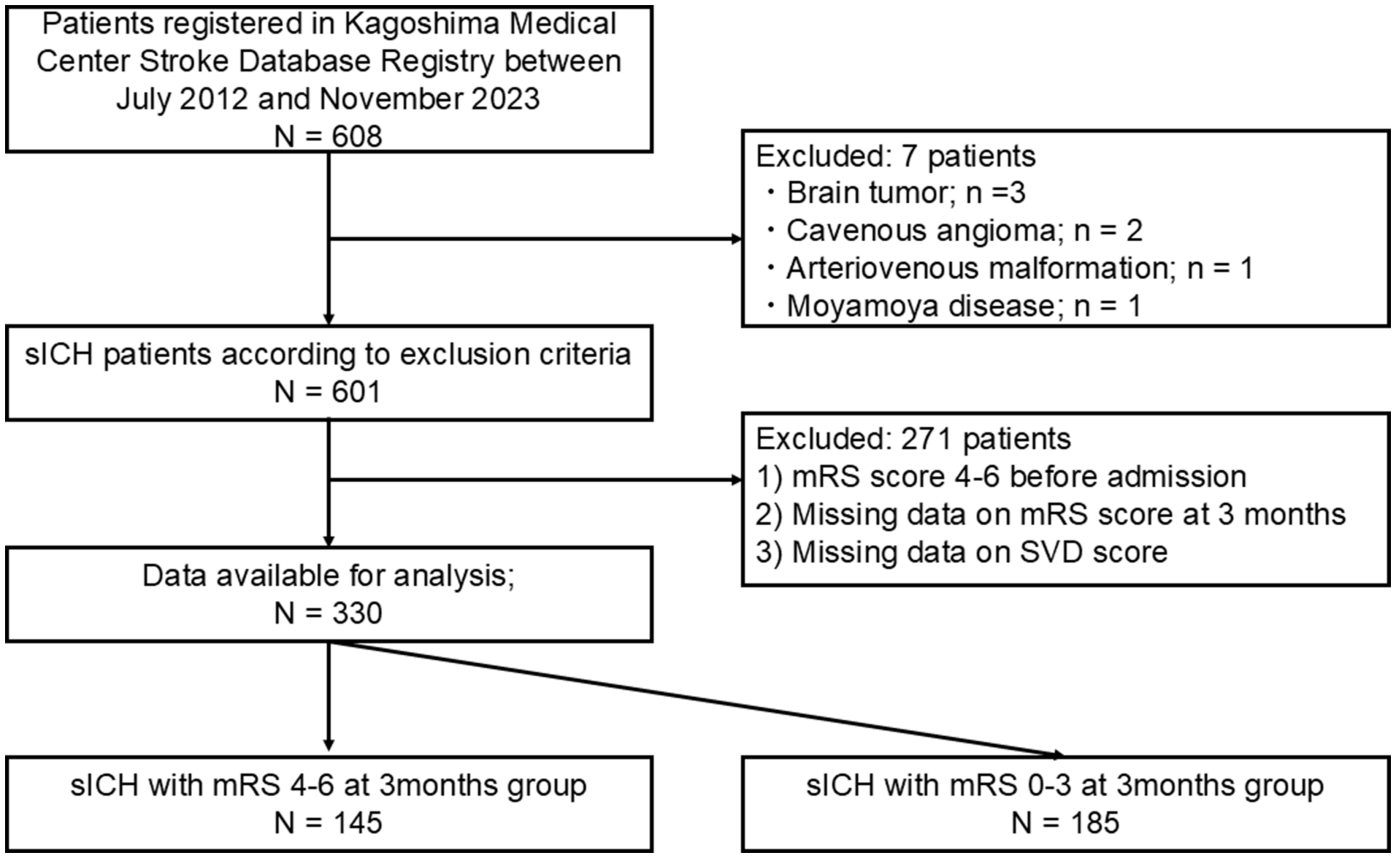
Flowchart of patient selection. sICH, spontaneous intracerebral hemorrhage; mRS, modified Rankin Scale; SVD, small vessel disease.

Figure 1 presents the study flowchart. Among the 608 patients initially considered, 330 were included in the analysis. At 3 months post-onset, 145 patients (43.9%) experienced poor outcomes, while 185 patients (56.1%) had favorable outcomes. In univariate analyses (Table 1), the prevalence of AF was significantly higher in the poor outcome group (20.7% vs. 9.2%, *p* = 0.004). Regarding AF subtypes, data were available only for paroxysmal and permanent AF. In the poor outcome group, 15 patients (10.3%) had permanent AF and 15 patients (10.3%) had paroxysmal AF, whereas in the favorable outcome group, six patients (3.2%) had permanent AF and 11 patients (6.0%) had paroxysmal AF. Data on persistent AF were not collected in this registry. The detailed distribution of cardiac comorbidities is shown in Supplementary material 1. Among patients with AF, structural cardiac disorders associated with arrhythmia were more frequently observed than isolated atrial dysrhythmia, and the distribution is also presented separately for men and women. Lobar ICH was observed in 32 patients (22.1%) in the poor outcome group and in 47 patients (25.4%) in the good outcome group, with no significant difference between the two groups (*p* = 0.517). In contrast, intraventricular hemorrhage was significantly more frequent in patients with poor outcomes compared with those with good outcomes (55.9% vs. 12.4%, *p* < 0.001).

**Table 1:** Comparison between patients with spontaneous intracerebral hemorrhage showing modified Rankin Scale (mRS) scores 4–6 and 0–3.

Variables	ICH with mRS 4–6 *N* = 145	ICH with mRS 0–3 *N* = 185	*p*-value
Age, y (mean ± SD)	76.2 ± 11.8	66.2 ± 13.8	<0.001
Male, *n* (%)	71 (49.0)	102 (55.1)	0.270
Prestroke mRS score, median (IQR)	0 (0–1)	0 (0–0)	0.001
Systolic BP, mmHg, (IQR)	172 (148–200)	170 (150–189)	0.474
Diastolic BP, mmHg, (IQR)	91 (78–108)	96 (81–109)	0.162
BMI, (IQR)	22.0 (19.9–24.1)	23.7 (21.3–26.7)	<0.001
Wake up stroke, *n* (%)	19 (13.3)	23 (12.6)	0.869
Current smoking, *n* (%)	19 (13.8)	36 (19.9)	0.179
Excessive drinking, *n* (%)	14 (10.1)	27 (15.0)	0.238
Baseline NIHSS score, median (IQR)	14 (9–19)	3 (2–8)	<0.001
Comorbidities			
Hypertension, *n* (%)	141 (97.2)	170 (91.9)	0.054
Dyslipidaemia, *n* (%)	51 (35.2)	81 (43.8)	0.141
Diabetes mellitus, *n* (%)	34 (23.5)	37 (20.0)	0.500
Atrial fibrillation, *n* (%)	30 (20.7)	17 (9.2)	0.004
Previous stroke, *n* (%)	42 (29.0)	41 (22.2)	0.162
Oral anticoagulation before admission, *n* (%)	25 (17.2)	27 (14.6)	0.545
Oral antiplatelet before admission, *n* (%)	36 (24.8)	39 (21.1)	0.430
Laboratory data			
Hemoglobin (g/dL)	13.1 (11.8–14.4)	14.1 (12.6–15.2)	<0.001
Hematocrit (%)	39.0 (35.3–42.8)	42.0 (37.9–44.8)	<0.001
Blood urea nitrogen (mg/dL)	16.5 (13.6–21.5)	15.4 (12.3–19.2)	0.007
Creatinine (mg/dL)	0.76 (0.58–0.94)	0.74 (0.58–0.96)	0.801
Uric acid (mg/dL)	5.4 (4.5–6.4)	5.3 (4.4–6.6)	0.784
Low density lipoprotein cholesterol (mg/dL)	101 (86–122)	116 (91–140)	0.004
Triglyceride (mg/dL)	91 (69–127)	103 (73–162)	0.059
Glycosylated hemoglobin (%)	5.7 (5.5–6.3)	5.8 (5.5–6.4)	0.647
Brain natriuretic peptide (pg/mL)	68.8 (26.3–168)	34.0 (16.6–79.0)	<0.001
D-dimer (μg/mL)	0.81 (0.43–2.05)	0.53 (0.32–1.06)	<0.001
Radiological characteristics			
Estimated hematoma volume, ml, median (IQR)	10.5 (5–21.7)	4.3 (1.5–9.7)	<0.001
Expansion of hematoma, *n* (%)	19 (13.6)	5 (2.7)	<0.001
Intraventricular hemorrhage, *n* (%)	81 (55.9)	23 (12.4)	<0.001
Hematoma involving left hemisphere, *n* (%)	58 (45.7)	74 (46.5)	0.905
Sites			
Lobar, *n* (%)	32 (22.1)	47 (25.4)	0.517
Putamen, *n* (%)	17 (11.7)	28 (15.1)	0.421
Thalamus, *n* (%)	76 (52.4)	82 (44.3)	0.151
Brainstem, *n* (%)	12 (8.3)	12 (7.0)	0.681
Cerebellum, *n* (%)	6 (4.1)	13 (7.0)	0.343
SVD markers			
Total SVD score, median (IQR)	2 (1–3)	2 (1–3)	0.021
PVH Fazekas, median (IQR)	1 (1–3)	1 (1–2)	<0.001
DSWMH Fazekas, median (IQR)	2 (1–3)	1 (1–2)	0.005
Total number of CMBs, median (IQR)	4 (1–10)	2 (1–7)	0.035
Number of CMBs in deep brain region, median (IQR)	1 (0–4)	1 (0–3)	0.909
Number of CMBs in lobar, median (IQR)	1 (0–3)	0 (0–2)	0.060
Number of CMBs in subtentorial, median (IQR)	1 (0–2)	0 (0–1.5)	0.089
Severe EPVS in basal ganglia, median (IQR)	1 (1–2)	1 (1–2)	0.027
Old lacunes, *n* (%)	64 (44.1)	73 (39.5)	0.431
Additional treatment			
The use of lyophilized human prothrombin complex concentrate, *n* (%)	0 (0)	0 (0)	N.A.
The use of idarucizumab, *n* (%)	0 (0)	0 (0)	N.A.
The use of andexanet alfa, *n* (%)	1 (0.7)	4 (2.2)	0.390
Operative surgery, *n* (%)	11 (6.6)	3 (2.1)	0.097

Data are presented as number (%) or median (interquartile range) values. Ages are shown as mean and standard deviation (SD) values. AF, atrial fibrillation; BMI, body mass index; BP, blood pressure; CMBs, cerebral microbleeds; DSWMH, deep and subcortical white matter hyperintensity; EPVS, enlarged perivascular spaces; mRS, modified Rankin Scale; NIHSS, National Institutes of Health Stroke Scale; PVH, periventricular hyperintensities; SVD, small vessel diseases. Forty-four cases of brainstem hemorrhage and cerebellar hemorrhage excluded.

Total SVD score was significantly higher in the poor outcome group [median 2 (IQR 1–3) vs. 2 (IQR 1–3), *p* = 0.021]. SVD markers were also significantly more prevalent in the poor outcome group, including PVH Fazekas [median 1 (IQR 1–3) vs. 1 (IQR 1–2), *p* < 0.001], DSWMH Fazekas [median 2 (IQR 1–3) vs. 1 (IQR 1–2), *p* = 0.005], total number of CMBs [median 4 (IQR 1–10) vs. 2 (IQR 1–7), *p* = 0.035], and severe EPVS in the basal ganglia [median 1 (IQR 1–2) vs. 1 (IQR 1–2), *p* = 0.027]. Use of andexanet alfa was reported in one case (0.7%) in the poor outcome group and four cases (2.2%) in the favorable outcome group, but the difference was not significant. Use of lyophilized human prothrombin complex concentrate or idarucizumab was not observed in any case. Among patients who underwent additional surgical interventions, 11 patients (6.6%) were in the poor outcome group and 3 patients (2.1%) were in the favorable outcome group. The breakdown included eight cases with craniotomy for hematoma evacuation, two cases with ventricular drainage, and one case who underwent both procedures in the poor outcome group, compared to three cases with craniotomy for hematoma evacuation in the favorable outcome group. Among the 23 patients who died within 3 months, 10 deaths were classified as neurological (acute hydrocephalus in three cases and brain herniation in seven cases), whereas 13 were classified as non-neurological causes. The latter included aspiration pneumonia (*n* = 5), heart failure (*n* = 2), and one case each of hepatic failure, respiratory failure, fatal arrhythmia, asphyxia, sepsis, and leukemia.

**Figure 2:**
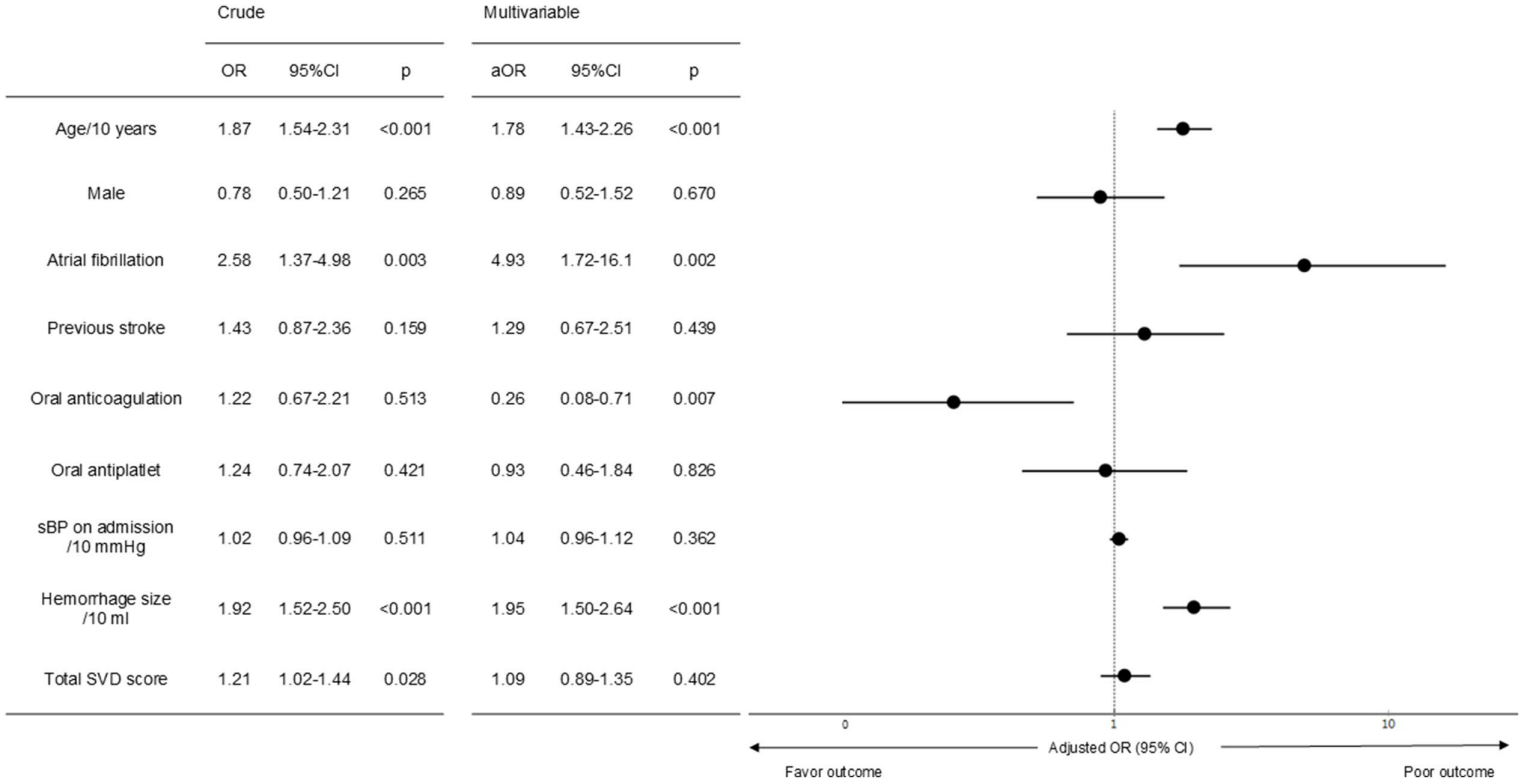
Logistic regression analysis of factors associated with poor outcome at 3 months, adjusted for age, sex, presence of atrial fibrillation, history of previous stroke, use of oral anticoagulants, use of oral antiplatelet agents, systolic blood pressure on admission, estimated hemorrhage size, total SVD score. CI, confidence interval; OR, odds ratio; sBP, systolic blood pressure; SVD, small vessel disease.

In the logistic regression analysis with poor outcome at 3 months as the dependent variable, presence of AF was independently associated with poor outcome [adjusted OR (aOR) 4.93, 95% CI 1.72–16.1; *p* = 0.002], while no significant association was observed with total SVD score (Figure 2). In the multivariate logistic regression model, the discriminatory performance was acceptable, with an area under the ROC curve (AUC) of 0.804. The model demonstrated a sensitivity of 71.4%, specificity of 77.2%, positive predictive value of 73.0%, negative predictive value of 72.4%, and an overall diagnostic accuracy of 72.7%. In a sensitivity analysis (Model 2) further adjusted for baseline mRS and NIHSS on admission, AF remained significantly associated with poor 3-month outcomes (adjusted OR 5.47, 95% CI 1.50–23.6; *p* = 0.009), whereas the total SVD score did not show a significant association (Supplementary material 2).

Similarly, in the multiple regression analysis with estimated hematoma volume as the dependent variable, AF showed a significant association (partial regression coefficient 8.18, 95% CI 0.65–15.7, standardized regression coefficient 0.16; *p* = 0.033), whereas total SVD score did not demonstrate a significant association (Table 2).

**Table 2:** Multiple regression analysis with estimated hematoma volume as an explanatory variable.

Variables	Partial regression coefficient	95% CI	*β*	VIF	*p*-value
Age	0.23	0.08–0.38	0.18	1.20	0.003
Male	0.47	−1.50 to 2.44	0.03	1.08	0.639
AF	8.18	0.65–15.7	0.16	1.93	0.033
Total SVD score	−1.12	−2.72 to 0.48	−0.08	1.17	0.168
Previous stroke	0.02	−2.51 to 2.54	0.01	1.34	0.989
Oral anticoagulation	1.36	−2.29 to 5.01	0.06	1.97	0.463
Oral antiplatelet	0.27	−2.32 to 2.86	0.01	1.31	0.840
sBP on admission	0.02	−0.04 to 0.08	0.04	1.05	0.478

Multivariable models were adjusted for age, sex, AF, total SVD score, previous stroke, oral anticoagulation, oral antiplatelet, sBP on admission. AF, atrial fibrillation; CI, confidence interval; mRS, modified Rankin Scale; sBP, systolic blood pressure; SVD, small vessel diseases; VIF, variance inflation factor; *β*, standardized partial regression coefficient.

**Figure 3:**
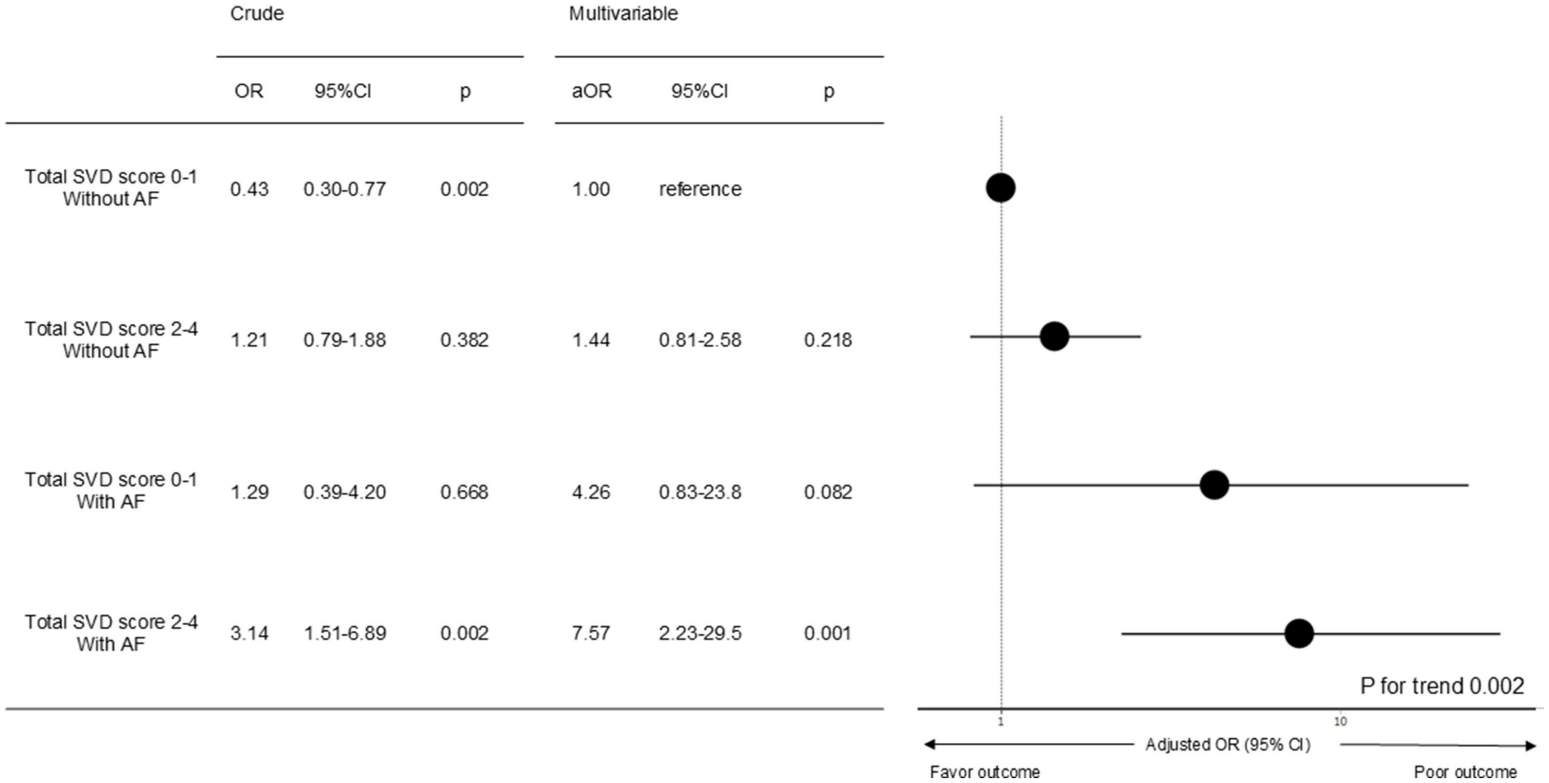
Multivariate analysis of poor prognosis using the presence or absence of atrial fibrillation and total SVD score severity as indicator variables. To confirm the linear association between the presence or absence of AF and total SVD score severity, patients were divided into 4 groups and each group was given an indicator variable (total SVD score 0–1 without AF: 1; total SVD score 2–4 without AF: 2; total SVD score 0–1 with AF: 3; and total SVD score 2–4 with AF: 4). Another logistic regression model with index variables was constructed, using total SVD score 0–1 without AF as the reference criterion, further adjusted for sex, age, and factors associated with poor outcome (history of stroke, oral anticoagulants, oral antiplatelet agents, systolic blood pressure on admission, and estimated hemorrhage size). AF, atrial fibrillation; aOR, adjusted odds ratio; CI, confidence interval; OR, odds ratio; SVD, small vessel disease.

The logistic regression analysis evaluating the combination of AF and total SVD score showed a linear trend, indicating an increased risk of poor outcomes in the group with AF and higher total SVD score. Using total SVD score 0–1 without AF as the reference, aORs were as follows: total SVD score 0–1 without AF, aOR 1.00 (reference); total SVD score 2–4 without AF, aOR 1.44 (95% CI 0.81–2.58, *p* = 0.218); total SVD score 0–1 with AF, aOR 4.26 (95% CI 0.83–23.8, *p* = 0.082); total SVD score 2–4 with AF, aOR 7.57 (95% CI 2.23–29.5, *p* = 0.001) (*p* for trend = 0.002) (Figure 3).

**Figure 4:**
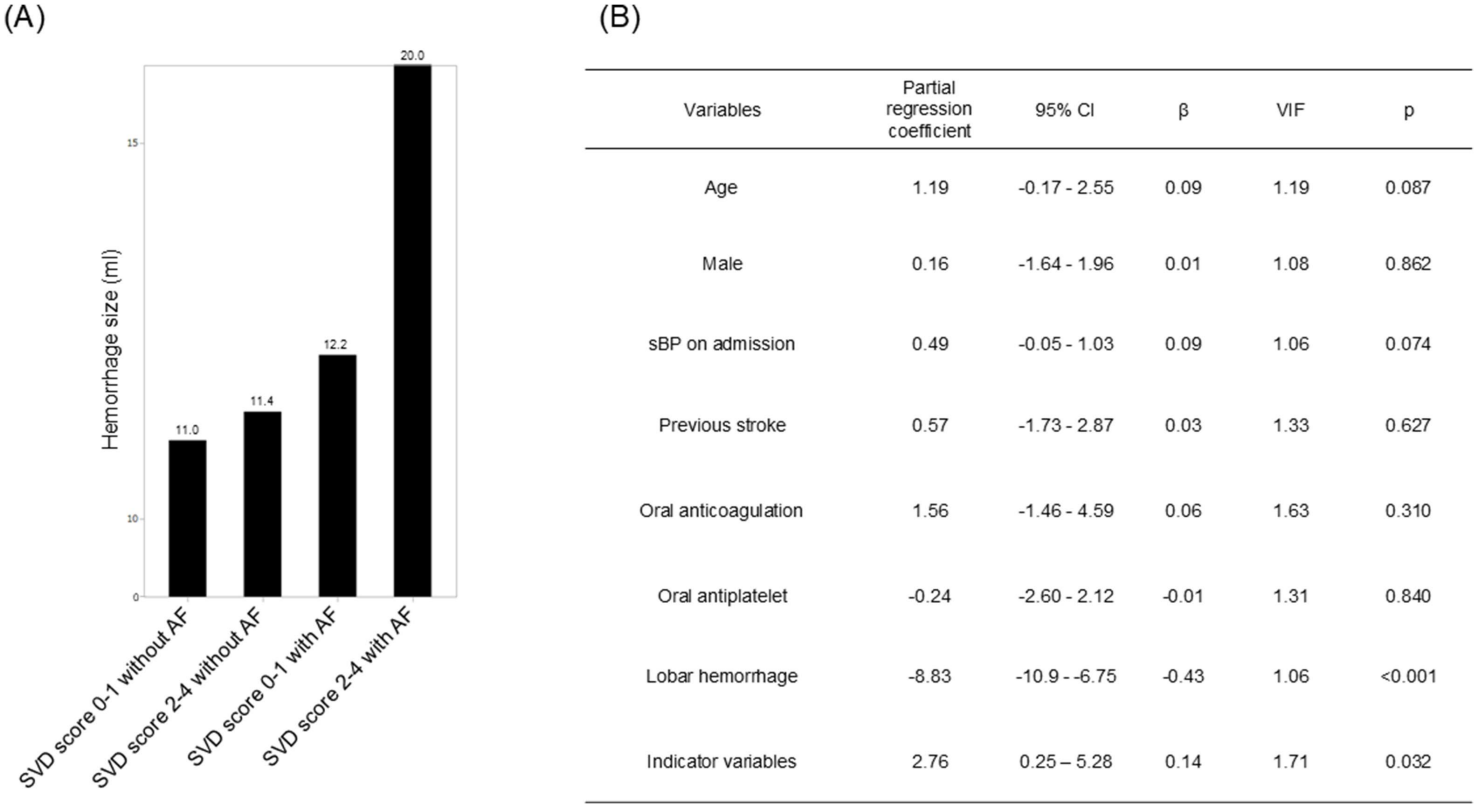
**(A)** Bar chart of mean estimated hematoma volume for each indicator variable. Presence or absence of atrial fibrillation and severity of total SVD score were divided into four groups, with each group given an indicator variable (total SVD score 0–1 without AF: 1; total SVD score 2–4 without AF: 2; total SVD score 0–1 with AF: 3; total SVD score 2–4 with AF: 4). **(B)** Multiple regression analysis of estimated hematoma volume with presence or absence of atrial fibrillation and total SVD score severity as indicator variables. A dummy variable with the indicator variable as an ordinal variable was given, and a multiple regression model was constructed including this dummy variable and further adjusted for sex, age, and factors associated with poor outcome (history of stroke, oral anticoagulants, oral antiplatelet agents, systolic blood pressure on admission, and presence of subcortical hemorrhage). AF, atrial fibrillation; aOR, adjusted odds ratio; CI, confidence interval; OR, odds ratio; SVD, small vessel disease; VIF, variance inflation factor; *β*, standardized partial regression coefficient.

Mean estimated hematoma volumes for each group were 11.0 mL for SVD score 0–1 without AF, 11.4 mL for SVD score 2–4 without AF, 12.2 mL for SVD score 0–1 with AF, and 20.0 mL for SVD score 2–4 with AF (Figure 4A). In addition, the multiple regression analysis that included the four groups divided by AF status and total SVD score severity as ordinal variables, presence of AF and higher SVD score correlated significantly with increased estimated hematoma volume (partial regression coefficient 2.76, 95% CI 0.25–5.28, standardized regression coefficient 0.14; *p* = 0.032) (Figure 4B).

## 4 Discussion

This study demonstrated the following findings. First, in this study of consecutive sICH patients, the presence of AF significantly increased the likelihood of poor outcomes at 3 months post-sICH, whereas the severity of SVD was not independently associated with prognosis. Second, estimated hematoma volume was associated with the presence of AF but was not independently related to SVD severity. Third, the combination of AF and a high total SVD score appeared to have a synergistic effect, contributing to both poor outcomes and increased hematoma volume. These findings suggest that SVD plays a modifying role in the relationship between AF and sICH, as well as in the pathophysiological mechanisms, providing novel insights.

OACs represent a well-known risk factor for sICH. While some reports have described the impact of OAC use on the prognosis of sICH patients with AF (8, 14), studies specifically investigating the impact of AF itself on sICH outcomes are scarce. Previous research has suggested that the severity of SVD contributes to the occurrence of sICH in patients with AF who are taking OACs (33), emphasizing that anticoagulant therapy alone should not be considered the sole cause of sICH (33). Moreover, recent evidence has further highlighted the role of AF in hemorrhagic complications. Ahmed et al. demonstrated that, in AF patients receiving thrombolysis for embolic stroke, specific clinical predictors were associated with different subtypes of hemorrhagic transformation (34). These findings support the concept that AF itself, beyond anticoagulant therapy, is an important determinant of hemorrhagic risk and outcomes, consistent with our observations in patients with sICH.

Indeed, AF has been reported to promote the progression of WMHs, which are a component of the total SVD score (35), suggesting a strong relationship between AF and WMHs. Pathological studies of WMHs have described diffuse vacuolation, spongiform changes, arteriosclerosis, tissue rarefaction due to myelin and axonal loss, EPVS, and decreased glial cell density (36). In particular, severe WMHs have been associated with increased brain tissue rarefaction and disruption of the blood–brain barrier (BBB), leading to larger ICH volumes and hematoma expansion (33).

The presence of AF may thus be linked to the progression of WMHs, leading to increased fragility of brain tissues and contributing to hematoma accumulation and poor sICH outcomes.

Further, estimated hematoma volume in this study was associated with the presence of AF. Regarding hematoma expansion, Fisher (37) proposed the “avalanche hypothesis,” which suggests that the mass effect of the initial hematoma may damage surrounding arteries and arterioles, leading to secondary hemorrhage. Recently, the first experimental evidence supporting Fisher’s avalanche hypothesis was reported using an experimental ICH model (38). In addition, irregular pulse waves associated with AF and disruption of the BBB may cause fluctuations in cerebral perfusion pressure, reducing counterpressure from surrounding tissues, which could contribute to hematoma expansion. A higher SVD score indicates greater fragility and rarefaction of the surrounding brain tissue, which may facilitate intraventricular extension once hematoma expansion occurs. Previous studies have reported that intraventricular hemorrhage is an important determinant of poor outcome, owing to its association with larger hematoma burden and secondary complications such as hydrocephalus (39). Consistent with these findings, intraventricular extension of ICH was significantly more frequent in patients with poor outcomes in our cohort. Analysis of the four groups stratified by AF status and total SVD score severity revealed a significant linear trend, with a higher risk of poor outcomes and greater estimated hematoma volume in the group with both AF and high total SVD score.

Interestingly, since total SVD score alone was not independently associated with poor sICH outcome or estimated hematoma volume, the synergistic effect of AF and SVD warrants attention. This suggests that SVD may play a catalytic role in worsening outcomes and increasing estimated hematoma volume among sICH patients with AF. As mentioned earlier, the presence of AF may contribute to increased initial hematoma volume by disrupting brain tissue integrity through the association with WMHs. Moreover, the higher the SVD score, the more fragile the brain tissue becomes, and the reduced counterpressure from surrounding tissues may facilitate infiltration of hemorrhage into adjacent areas, potentially leading to increases in hemorrhage volume.

In ischemic stroke, the cumulative effect of SVD markers is known to influence poor outcomes (40), and individual SVD markers such as WMHs, CMBs, and EPVS have also been associated with gait disturbances (41). WMHs predominantly manifest in subcortical white matter, the centrum semiovale, and corona radiata, which contain the corticospinal tract responsible for voluntary motor commands. CMBs can affect gait function by preferentially damaging brain regions involved in gait control, such as the frontal lobe and basal ganglia. The severity of EPVS in the basal ganglia may impair basal ganglia function, leading to cumulative damage to connecting fibers with the frontal lobe and subsequently resulting in executive dysfunction (42). Executive dysfunction can contribute to gait disturbances from multiple perspectives. These findings suggest that the cumulative effects of SVD, along with the presence of AF, may collectively contribute to poor sICH outcomes.

One strength of this study was that estimated hematoma volume was measured using head CT at the time of admission, potentially reflecting the early-phase ICH volume. In addition, SVD markers were assessed using MRI obtained within 24 h of hospitalization. This approach allowed simultaneous evaluation of the relationship between ICH and WMHs at the same time point.

Further, this study targeted patients from the western region of Japan, with a relatively older population, making the results more applicable to real-world clinical settings. Findings in this study have significant implications from a preventive medicine perspective, highlighting the importance of SVD prevention in patients with AF. Translational research is warranted to develop pharmacological strategies to halt the progression of SVD, and prospective multicenter studies integrating radiological, pathological, and biomarker-based approaches are needed to provide more comprehensive insights and to establish novel preventive and therapeutic strategies.

### 4.1 Limitations

This study showed several limitations. First, a retrospective study design was applied to data collected from a single medical center from only East Asian patients. This would have influenced the results, potentially compromising both internal and external validity and limiting the generalizability of the findings. In addition, although our registry recorded a “history of any stroke,” including ischemic stroke, intracerebral hemorrhage, and TIA, TIA was not collected as an independent variable, and thus its frequency could not be reported separately. Moreover, several potential confounders—such as elevated body temperature at presentation, medication adherence, adherence to physiotherapy or rehabilitation, and early blood pressure variability—were not available in our registry and therefore could not be adjusted for in the analysis. These unmeasured variables may have influenced the observed associations. Otherwise, we found that among sICH patients with AF, those with higher WMHs scores tended to show poorer discharge outcomes compared to those with lower scores. However, we did not track long-term follow-up data for most patients, preventing assessment of the impact of WMHs on long-term functional outcomes and mortality. Further, hematoma volume was estimated only at the time of hospital admission, without continuous measurements. As a result, the temporal relationship between sICH and hematoma volume, as well as the impacts on morbidity and mortality, may lack precision. Future prospective studies should address these limitations.

## 5 Conclusion

The presence of AF was associated with poor outcomes at 3 months and increased hematoma volume compared to sICH patients without AF. While total SVD score alone did not significantly impact poor outcomes or hematoma volume, the combination with AF exhibited a synergistic effect leading to increases in both poor outcomes and hematoma volume. These findings contribute to the understanding of the pathophysiological mechanisms in AF-related sICH and may aid in developing preventive strategies for sICH in patients with AF.

## Data Availability

The original contributions presented in the study are included in the article/Supplementary material, further inquiries can be directed to the corresponding author.
