# Supplementary material for: The combination of atrial fibrillation and small vessel disease score worsen spontaneous intracerebral hemorrhage outcomes
Source: Front Neurol. 2025 Oct 29;16:1682520. doi: 10.3389/fneur.2025.1682520 (PMC12605216; doi:10.3389/fneur.2025.1682520)
Supplement: Supplementary file 1 [file Table_1.docx]

Supplementally material 1. Cardiac comorbidities in patients with atrial fibrillation.

| Cardiac comorbidity | AF total (n=47) | Men  (n=33) | Women (n=14) |
| --- | --- | --- | --- |
| Structural cardiac disorders associated with arrhythmia, n (%)¶ | 37 (84.1) | 26 (86.7) | 11 (78.6) |
| Valvular heart disease, n (%)§ | 25 (59.5) | 16 (57.1) | 9 (64.3) |
| Cardiomyopathy, n (%)§ | 0 | 0 | 0 |
| Ischemic heart disease, n (%)§ | 11 (23.4) | 7 (21.2) | 4 (28.6) |
| Left ventricular hypertrophy, n (%)§ | 24 (57.1) | 17 (60.7) | 7 (50.0) |
| Left ventricular dysfunction, n (%)§ | 24 (57.1) | 15 (53.6) | 9 (64.3) |
| Isolated atrial dysrhythmia, n (%)¶ | 7 (15.9) | 4 (13.3) | 3 (21.4) |

AF (Atrial fibrillation) patients were categorized according to Capmany et al. [20] into two groups: (a) structural cardiac disorders associated with arrhythmia (e.g., valvular heart disease [VHD], cardiomyopathy, ischemic heart disease [IHD], left ventricular hypertrophy [LVH, defined as interventricular septum or posterior wall thickness ≥12 mm in men or ≥11 mm in women on echocardiography], and left ventricular dysfunction [LVD, defined as reduced ejection fraction <50% or abnormal diastolic parameters]); and (b) isolated atrial dysrhythmia (AF without structural heart disease). The subcategories of structural cardiac disorders were not mutually exclusive, and some patients had overlapping conditions. ¶: 3 missing values; §: 5 missing values.
Abbreviations: AF, atrial fibrillation; VHD, valvular heart disease; IHD, ischemic heart disease; LVH, left ventricular hypertrophy; LVD, left ventricular dysfunction.
