# Supplementary material for: The combination of atrial fibrillation and small vessel disease score worsen spontaneous intracerebral hemorrhage outcomes
Source: Front Neurol. 2025 Oct 29;16:1682520. doi: 10.3389/fneur.2025.1682520 (PMC12605216; doi:10.3389/fneur.2025.1682520)
Supplement: Supplementary file 2 [file Table_2.docx]

Supplementally material 2. Multivariable logistic regression analysis of factors associated with poor outcomes at 3 months (Model 2).

| Variables | Adjusted odds ratio | 95% CI | P value |
| --- | --- | --- | --- |
| Age/10 years | 1.86 | 1.43-2.46 | <0.001 |
| Male | 1.03 | 0.56-1.92 | 0.921 |
| Atrial fibrillation | 5.47 | 1.50-23.6 | 0.009 |
| Previous stroke | 0.96 | 0.42-2.19 | 0.931 |
| Oral anticoagulation | 0.30 | 0.07-1.08 | 0.067 |
| Oral antiplatelet | 1.25 | 0.55-2.80 | 0.592 |
| sBP on admission/10 mmHg | 1.03 | 0.94-1.13 | 0.540 |
| Hemorrhage size/10 mmHg | 1.34 | 1.03-1.82 | 0.027 |
| Total SVD score | 1.10 | 0.86-1.42 | 0.433 |
| mRS on admission | 1.45 | 0.96-2.24 | 0.078 |
| NIHSS score on admission | 1.24 | 1.17-1.32 | <0.001 |

Model 2: Logistic regression analysis of factors associated with poor outcome at 3 months, adjusted for age, sex, presence of atrial fibrillation, history of previous stroke, use of oral anticoagulants, use of oral antiplatelet agents, systolic blood pressure on admission, estimated hemorrhage size, total SVD score, mRS on admission, and NIHSS score on admission. CI, confidence interval; mRS, modified Rankin Scale; NIHSS, National Institutes of Health Stroke Scale; OR, odds ratio; sBP, systolic blood pressure; SVD, small vessel disease.
